# A Common Polymorphism of the Human Cardiac Sodium Channel Alpha Subunit (SCN5A) Gene Is Associated with Sudden Cardiac Death in Chronic Ischemic Heart Disease

**DOI:** 10.1371/journal.pone.0132137

**Published:** 2015-07-06

**Authors:** Boglárka Marcsa, Réka Dénes, Krisztina Vörös, Gergely Rácz, Mária Sasvári-Székely, Zsolt Rónai, Klára Törő, Gergely Keszler

**Affiliations:** 1 Department of Medical Chemistry, Molecular Biology and Pathobiochemistry, Semmelweis University, Budapest, Hungary; 2 Department of Forensic and Insurance Medicine, Semmelweis University, Budapest, Hungary; 3 1st Department of Pathology and Experimental Cancer Research, Semmelweis University, Budapest, Hungary; Johns Hopkins University, UNITED STATES

## Abstract

Cardiac death remains one of the leading causes of mortality worldwide. Recent research has shed light on pathophysiological mechanisms underlying cardiac death, and several genetic variants in novel candidate genes have been identified as risk factors. However, the vast majority of studies performed so far investigated genetic associations with specific forms of cardiac death only (sudden, arrhythmogenic, ischemic etc.). The aim of the present investigation was to find a genetic marker that can be used as a general, powerful predictor of cardiac death risk. To this end, a case-control association study was performed on a heterogeneous cohort of cardiac death victims (n=360) and age-matched controls (n=300). Five single nucleotide polymorphisms (SNPs) from five candidate genes (beta2 adrenergic receptor, nitric oxide synthase 1 adaptor protein, ryanodine receptor 2, sodium channel type V alpha subunit and transforming growth factor-beta receptor 2) that had previously been shown to associate with certain forms of cardiac death were genotyped using sequence-specific real-time PCR probes. Logistic regression analysis revealed that the CC genotype of the rs11720524 polymorphism in the SCN5A gene encoding a subunit of the cardiac voltage-gated sodium channel occurred more frequently in the highly heterogeneous cardiac death cohort compared to the control population (p=0.019, odds ratio: 1.351). A detailed subgroup analysis uncovered that this effect was due to an association of this variant with cardiac death in chronic ischemic heart disease (p=0.012, odds ratio = 1.455). None of the other investigated polymorphisms showed association with cardiac death in this context. In conclusion, our results shed light on the role of this non-coding polymorphism in cardiac death in ischemic cardiomyopathy. Functional studies are needed to explore the pathophysiological background of this association.

## Introduction

Though the past decades have seen a rapid development in the field of therapeutic options, sudden cardiac death (SCD) still remains a leading cause of mortality worldwide [1]. The underlying pathological processes—acute or chronic heart failure—might be elicited by numerous factors including coronary artery disease, hypertension, valvular dysfunctions, primary cardiomyopathy, inflammatory diseases as well as arrhythmias. In patients above 50 years of age, coronary heart disease and concomitant chronic ischemic cardiomyopathy account for 75–80% of SCD cases [2]. Persistent myocardial ischemia might elicit ventricular tachyarrhytmias via several mechanisms [3]. On the one hand, myocardial hypoxia is a potent arrhythmogenic factor that impairs ionic homeostasis by blocking ion pumps and thereby interferes with cardiac ion channel conductance in the plasma membrane [3]. Moreover, the emergence of arrhythmogenic foci in myocardial remodeling compromises the electrical activity of the heart, playing a causative role in the occurrence of fatal arrhythmias that underlie sudden decompensation and cardiac death [4, 5]. Genetic markers governing structural remodeling include a large body of genetic variants that impair force generation, calcium cycling and transcriptional regulation of cardiac gene expression [6] as well as perturbed expression of regulatory microRNAs [7].

Recently, numerous genetic studies have been conducted to identify candidate genes whose mutations and polymorphisms are associated with increased cardiovascular risk in general and sudden cardiac death in particular. Notably, these prospective investigations have shed light on the seminal role of rare and common variants of genes encoding cardiac voltage-gated ion channels, plasma membrane receptors and intracellular signaling proteins [8–11].

As far as channelopathies are concerned, the SCN5A gene encoding the α subunit of the cardiac voltage-gated sodium channel (Nav1.5) might harbor several mutations associated with arrhythmia vulnerability syndromes including the Brugada syndrome [12], sick sinus syndrome [13], the arrhythmogenic long QT syndrome [14] and susceptibility to ventricular fibrillation during myocardial infarction [15]. Though the vast majority of SCN5A mutations elicit impaired protein structure, common promoter or intronic single nucleotide polymorphisms of the SCN5A gene can also associate with arrhythmia and sudden cardiac death [16–19].

Mutations and common polymorphic variants of the type 2 ryanodine receptor, a heart-specific sarcoplasmic reticulum calcium release channel, have also been implicated in certain cardiac electrophysiological disorders such as catecholaminergic polymorphic ventricular tachycardia and familial arrhythmogenic ventricular dysplasias [20–22] and sudden cardiac death.

Though first described as a neuron-specific gene governing neuronal signaling and synaptic plasticity, the nitric oxide synthase 1 adaptor protein (NOS1AP) has also been shown to modulate the intracellular calcium homeostasis in cardiomyocytes [23]. In light of this, it is not surprising that polymorphic variants of the gene are associated with long QT syndrome and ensuing fatal arrhythmias [24, 25] as well as increased cardiovascular mortality among patients taking calcium channel blockers [26].

Myocardial hypertrophy, fibrosis and arterial wall remodeling are governed chiefly by members of the TGFβ signaling pathway. In accordance with this, pathological roles have been ascribed to variants of the transforming growth factor beta receptor 2 (TGFBR2) in aortic aneurysm [27], Kawasaki disease [28] and sudden cardiac arrest against the background of coronary artery disease [29]. Finally, polymorphisms of the type 2 adrenergic receptor, a well-known obesity and metabolic syndrome susceptibility gene that mediates sympathetic activation in various tissues, have been implicated in increased mortality of patients with cardiovascular disease [30–32] in general and sudden cardiac death in particular [31].

The aim of the present case-control study was to find genetic polymorphisms that might be independent and universal predictors of sudden cardiac death. To this end, selected single nucleotide polymorphisms occurring in the above mentioned candidate genes have been genotyped in a cohort of Hungarian patients who succumbed to different cardiovascular ailments.

## Materials and Methods

### Subjects

Buccal swabs from 360 victims of natural, cardiovascular death (66.7% male, mean age: 68.02 ± 14.45 years) were collected *post mortem* during autopsies at the Department of Forensic and Insurance Medicine (N = 262) and the 1st Department of Pathology (N = 98) of the Semmelweis University between Sept 2011 and Nov 2013. Regarding the circumstances of death, 165 cases (45.8%) were witnessed, unexpected, ‘established SCDs’ occurring within an hour of symptom onset: 28 in hospital, 17 in public institutions, 37 in ambulance and 83 in urban public places. 195 unwitnessed cases (54.2%) occurred at home without obvious extracardiac cause, within 24 hours of last being observed in good, symptom-free condition, therefore fulfilling the internationally acknowledged criteria of ‘probable SCD’ [33].

Buccal sampling was carried out within four days after death on the average. Morphological inclusion criteria were valvular heart disease; myocardial hypertrophy, fibrosis, fatty degeneration or atrophy; calcification or thrombus in the coronary arteries; moderate or severe atherosclerosis in other arteries; and embolism in the pulmonary arteries. The 10^th^ revision of the International Classification of Diseases (ICD) was used for categorizing the cause of death (Table 1). Violent death cases and cases where cardiovascular complications were consequences of underlying primary diseases (such as malignancies and pneumonias) were excluded from the study. No clinical records or data on medication, smoking, lifestyle factors etc. were considered.

**Table 1:** Characterization of the case population according to ICD (International Classification of Diseases) diagnoses and gender distribution.

	ICD Codes	Total		Males		Females	
		N	%	N	%	N	%
**Acute myocardial infarction**	I210-249	34	9.5%	21	8.8%	13	10.8%
**Chronic ischemic heart disease**	I250-259	228	63.3%	165	68.7%	63	52.5%
**Pulmonary embolism**	I260-269	25	7.0%	13	5.4%	12	10.0%
**Nonrheumatic valve disorders**	I340-359	7	1.9%	5	2.1%	2	1.7%
**Myocardial degeneration and cardiomegaly**	I515-517	59	16.4%	31	12.9%	28	23.3%
**Aortic aneurysm rupture or dissection**	I710-729	7	1.9%	5	2.1%	2	1.7%
		360	100.0%	240	100.0%	120	100.0%

The age-matched control group comprised 300 non-related volunteers (39.3% male, mean age: 65.75 ± 14.83 years) without any known cardiovascular disease. Buccal samples of the participants were collected at the Institute of Psychology, Eötvös Loránd University, Budapest, Hungary.

All case and control subjects were Caucasian whites from the administrative area of Budapest.

### SNP selection

Of a multitude of polymorphisms known to associate with SCD, five single nucleotide polymorphisms (SNPs) were selected from the candidate genes described in the Introduction. Selection of marker SNPs was based on results of previous genetic studies (Table 2).

**Table 2:** Results from individual studies used for SNP selection in the present study.

Gene	SNP and position	Study	Phenotype
SCN5A	rs11720524 [C/G] intron 1	Albert et al; 2010 [18]	Associated with cases of sudden and/or arrhytmic cardiac death in individuals of European ancestry
RYR2	rs790896 [A/G] 3′-UTR	Ran et al; 2010 [22]	Associated with sudden cardiac death in patients with chronic heart failure in Chinese Han population
ADRB2	rs1042714 [C/G] exon 1	Kulminski et al; 2010 [32]	Associated with the incidence of myocardial infarction in Framingham Heart Study Offspring cohort
TGFBR2	rs9838682 [A/G] intron 3	Tseng et al; 2009 [29]	Associate with sudden cardiac arrest in Caucasian patients with coronary artery disease
NOS1AP	rs10494366 [G/T] intron 1	Aarnoudse et al; 2007 [34]	Associated with the QT interval duration in Rotterdam Study cohort

HR = hazard ratio; OR = odds ratio.

### Genotyping

Handling of buccal samples and isolation of genomic DNA was performed essentially as described elsewhere [35]. Two parallel samples were obtained and processed from each participant. Concentration of genomic DNA solutions was measured by the AccuBlue Broad Range dsDNA Quantification kit (Biotium, Hayward, USA). Concentrations of DNA stock solutions varied between 15–200 ng/mL. Indicators of DNA purity were acceptable (OD 260/280 ratios were between 1.6–2.0 and OD 260/230 ratios varied between 2.0–2.2), and agarose gel electrophoretic assays revealed no significant fragmentation of genomic DNA.

1 μL samples from tenfold diluted stock solutions were used for genotyping.

Genotyping of selected single nucleotide polymorphisms (SNPs) was performed by a 7300 Real-Time PCR System (LifeTechnologies, USA) using commercially available TaqMan Genotyping Assays (Applied BioSystem, Foster City, USA), according to the manufacturer’s instructions. The following TaqMan assays were used: C_2084765_20 for the rs1042714 SNP in the ADRB2 gene; C_1777074 for rs10494366 in NOS1AP; C_409886_10 for rs790896 in RYR2; C_30666704_10 for rs11720524 in SCN5A and C_11907338_10 for rs9838682 in TGFBR2.

### Statistical analyses

Chi-square test of SPSS 17.0 for Windows was used for assessing the Hardy-Weinberg equilibria. A genotype-based logistic regression model adjusting for age and sex was used for case-control analysis. The Bonferroni correction was used for multiple testing. The Alibaba 2.1 transcription factor binding prediction software and the TRANSFAC database were employed for *in silico* transcription factor binding analysis.

### Ethics statement

The study protocol was designed in accordance with guidelines of the Declaration of Helsinki. Written informed consent was provided by all members of the control group. In accordance with the current Hungarian National Health Law (law No. CLIV/1997), however, there is no need to obtain informed consent from deceased individuals or their close relatives for *post mortem* investigations including DNA sampling and genotyping if the individual did not issue a declaration prohibiting any *post mortem* investigations before his/her death. To our best knowledge, none of the participants issued such written declarations that would have prevented them from being enrolled in the study.

The study design, the use of human samples and the above described consent procedure were all approved by the Scientific and Research Ethics Committee of the National Medical Research Council (ETT-TUKEB; permission number: ETT TUKEB-398/2013). Approval from the Institutional Review Board (IRB) was not necessary as the IRB is subordinate to ETT-TUKEB; however, the local IRB has also been informed on the issue of the ethical permission.

## Results

In order to find a genetic polymorphism that could be used as a universal predictor of cardiac death risk, we set up an intentionally very heterogeneous cohort of subjects who died of different cardiovascular illnesses as verified through autopsy. Table 1 demonstrates the stratification of the case population according to death causes. Though the majority of patients deceased of chronic myocardial ischemia, degeneration or hypertrophy, there were pronounced gender differences, as twice as many women died of pulmonary embolism and myocardial degeneration, while the diagnosis of chronic coronary heart disease was made more frequently among male subjects.

To dispel concerns on the quality of DNA obtained from *post mortem* sampling, we performed PCR-based genotyping of a well-known length polymorphism in the type 4 dopamine receptor gene that had much been studied previously in our laboratory [36]. Control PCR assays were run successfully on all *post mortem* samples, confirming that they were applicable for genetic testing

Five candidate genes of cardiac death have been selected from the current literature and one representative single nucleotide polymorphism from each gene was genotyped by quantitative PCR using allele-specific TaqMan probes. Though the list of genotyped variants is far from being comprehensive, the selected candidate genes are known to be critically involved in the pathomechanisms of sudden cardiac death which encompass dysrhythmias (SCN5A), perturbed intracellular calcium signaling (RYR2 and NOS1AP), myocardial remodeling (TGFBR2) and sympathetic activation (ADRB2). Accordingly, four SNPs analyzed in the present study (in genes ADRB2, RYR2, SCN5A and TGFBR2) have reportedly been associated with SCD (Table 2). The rs10494366 SNP in the NOS1AP gene showed strong association with cardiovascular mortality and high hazard ratio in patients treated with dihydropyridine calcium channel blockers. Here, we wished to address the issue whether this variant is associated with cardiac death in an extended, more heterogeneous patient population. Importantly, all genotype frequencies were in Hardy-Weinberg equilibrium in both the control and case population, and call rates were higher than 95% for each SNP investigated (data not shown).

Genotype distributions for each SNP in both populations as well as results of the case-control association study are presented in Table 3. Genotype frequencies were compared using a logistic regression model-based multivariate analysis accounting for age and gender differences between the case and control populations (Table 3). P values and odds ratios (OR) with confidence intervals (CI) for all five polymorphisms are shown in Table 3. Our results revealed that the CC genotype of the rs11720524 (G/C) SNP of the SCN5A gene occurred more frequently in the cardiovascular death cohort compared to the control group (47.90% vs. 36.99%, p = 0.019, OR = 1.351), while none of the other studied polymorphisms showed any significant differences in this respect.

**Table 3:** Case-control analysis of allele distributions.

Gene	dbSNP No.	alleles		control		case			95% CI	95% CI
			N	frequency	N	frequency	p	OR*	lower	upper
**ADRB2**	rs1042714	CC	106	36.68	132	37.18	0.868	1.020	0.810	1.283
		CG	133	46.02	161	45.35				
		GG	50	17.30	62	17.46				
**NOS1AP**	rs10494366	GG	40	14.34	50	14.62	0.625	1.061	0.836	1.347
		GT	129	46.24	160	46.78				
		TT	110	39.43	132	38.60				
**RYR2**	rs790896	AA	56	18.86	65	18.31	0.945	1.008	0.801	1.269
		AG	141	47.47	173	48.73				
		GG	100	33.67	117	32.96				
**SCN5A**	rs11720524	CC	108	36.99	171	47.90	**0.019**	1.351	1.050	1.737
		CG	149	51.03	155	43.42				
		GG	35	11.99	31	8.68				
**TGFB2**	rs9838682	AA	31	10.88	49	13.84	0.999	1.000	0.786	1.272
		AG	139	48.77	144	40.68				
		GG	115	40.35	161	45.48				

*OR = odds ratio; CI = confidence intervals.

Due to performing multiple comparisons, the accepted level of significance was corrected to avoid false positive results. After applying the stringent Bonferroni correction for multiple testing (p = 0.01 [0.05/5] as 5 SNPs were studied) the effect of the CC allele turned out to be slightly below the threshold of statistical significance.

The heterogeneity of the case population prompted us to perform a *post hoc* sub-analysis to figure out which case subgroup was responsible for the above effect. It turned out that the CC genotype occurred significantly more frequently in patients with chronic ischemic heart disease (p = 0.012, OR = 1.455) but not in the other patient subgroups (Table 4). As the statistical strength of the association was fairly enhanced by eliminating cases other than chronic ischemic heart disease, and the other SCD cases did not associate with the variant at all (p = 0.277), our data support the notion that the common rs11720524 CC variant is associated with increased SCD risk in patients with ischemic cardiomyopathy only, but not with the risk of SCD in general.

**Table 4:** Association of the SCN5A rs11720524 CC genotype with specific death causes in the case population.

Cause of death			95% CI	95% CI
	p	OR	lower	upper
acute myocardial infarction	0.198	1.468	0.818	2.633
chronic ischemic heart disease (IHD)	**0.012**	**1.455**	1.088	1.947
pulmonary embolism	0.790	1.093	0.569	2.099
nonrheumatic valve disorders	0.515	1.504	0.440	5.136
myocardial degeneration and cardiomegaly	0.939	1.017	0.661	1.565
aortic aneurysm rupture or dissection	0.188	2.701	0.616	11.854
non-IHD death cases	0.277	1.201	0.863	1.671

## Discussion

Given the high incidence and mortality of cardiovascular diseases, several prospective investigations have recently been initiated with the aim to identify genetic and epigenetic markers that predispose to cardiac death. Most association studies published so far have focused on a clinically well-characterized subgroup of patients to uncover genetic variants in key candidate genes [8, 12, 14, 15, 18, 19, 22–25]. Here we selected five broadly studied SNPs which are reportedly associated with certain forms of cardiac death and addressed the issue whether any of these genetic markers are of general and independent predictive value in assessing the risk of sudden cardiac death. In line with that, a particularly heterogeneous cohort of cardiac death victims was investigated irrespective of additional factors including co-morbidities, smoking and medication. Importantly, cardiac death victims were included on a random basis, without any bias, from individuals autopsied at the institutions specified above.

*Post mortem* DNA sampling for genetic studies is a rare but not unusual procedure, and there are reports claiming that the integrity of DNA in soft tissues is maintained even at ambient temperatures for several days after death [37]. Corpses involved in our study were refrigerated at 4°C before autopsy, and the quality of genomic DNA was carefully checked by spectrophotometry, agarose gel electrophoresis and control PCR assays.

Our results revealed that CC variant of the rs11720524 [C/G] intronic polymorphism in the SCN5A gene is associated with sudden cardiac death, but the association did not prove to be statistically significant upon correction for multiple testing. This fact prompted us to assume that there must be a more specific association between the gene variant and one (or some) of the SCD subgroups. To clarify this issue, a *post hoc* analysis was performed that shed light on a significant association of the CC genotype with the chronic ischemic heart disease cohort. The other SCD cohorts did not show association with this variant either alone or in combination (Table 4). Moreover, attenuation of the statistical power of the association by inclusion of the non-ischemic cohorts (p = 0.012 vs. 0.019, OR = 1.351 vs. 1.455) also supported the conclusion that the CC variant is associated with elevated SCD risk in chronic ischemic heart disease only but not with SCD in general.

The lack of statistical association of SCD in any individual non-ischemic cohorts with the gene variant might be attributable to the fact that sample sizes in these cohorts were very small, i. e. not commensurate to those in the chronic ischemic group that made out nearly two third of our SCD samples. A replication of the present study with bigger sample sizes might help elucidate this issue.

The direction of the effect of the rs11720524 SNP is similar to that observed in two previous studies. Albert et al. [18] described for the first time an association between the rs11720524 C allele and arrhythmogenic and ischemic sudden cardiac death (p = 0.0005, OR = 1.30). In their prospective study, 536 sudden cardiac death cases among US whites were included under very rigorous inclusion criteria (cardiac arrest occurred within 1 hour of symptom onset as documented by medical or next-of-kin reports), and cardiac risk factors such as smoking, diabetes, hypertension, hypercholesterolemia and family history were meticulously included in their statistical analysis. In our study, we included both established and probable ischemic cardiac death cases, and no cardiac risk factors were known or considered. This difference as well as the smaller sample size (N = 360) and the fact that they used an allele-wise conditional logistic regression analysis might explain why we obtained less significant association between the analyzed polymorphism and SCD. On the other hand, it is to note that our effect size (OR = 1.455) is outstandingly high for a common gene variant.

The predictive value of the same polymorphism has also been addressed by Son et al. [19] in the Korean population where—in contrast to Caucasian people—the C variant is the minor allele. Despite that, they found that the allele frequency of the C allele was twice as high in ventricular arrhythmia-based sudden cardiac death cases as in the control population. Although their results lack any statistical significance due to extremely small sample sizes (N = 14, p = 0.25, OR = 2.13), it confers further support on our notion that the predictive value of the C allele might be extended to other specific causes of sudden cardiac death if larger cohorts were analyzed.

The functional significance of the rs11720524 polymorphism remains to be elucidated. Nevertheless, our *in silico* analysis revealed that the presence of the protective G allele creates a DNA binding site for the POU domain containing transcription factor Oct-1. Though this binding site is localized in intron 1 of the gene, it is not uncommon that transcription factors are recruited to intronic regulatory sequences to confer enhanced transcription levels [38]. In light of that, it is tempting to speculate that SCN5A expression levels might be lower in patients harboring the CC genotype, and reduced SCN5A promoter activities have already been reported to predispose to arrhythmogenic conductivity disorders such as longer PR and QRS durations [39, 40]. This speculative assumption could explain the specific association of the CC variant with increased SCD frequency just in chronic ischemic heart disease. Hypoxia induced myocardial fibrosis is arrhythmogenic [3–5], and low SCN5A expression levels would further promote the emergence of life-threatening ventricular arrhythmias.

Furthermore, one cannot exclude the possibility that genetic variants of the SCN5A gene might influence expression levels via modulation of DNA methylation as proposed by Park et al. recently [16]. Last but not least, the rs11720524 marker SNP might be genetically coupled to other, functionally more important polymorphisms of the gene. To clarify this issue, a more detailed genotyping of several polymorphisms covering the 5’ and 3’ regulatory regions of the SCN5A gene should be performed.

Though the rest of investigated polymorphisms have previously been shown to associate with specific forms of sudden cardiac death [22, 26, 27, 30 – 32], we could not recapitulate these results in our highly heterogeneous case population probably due to low sample size.

Taken together, limitations of the study include the relatively small sample size, lack of replication on an independent case population, and lack of data on the impact of the rs11720524 alleles on gene expression. A more detailed analysis of other SCN5A polymorphisms should also be performed.

The major strength of the study presented here is demonstration that the homozygous CC genotype of the SCN5A rs11720524 SNP is significantly associated with SCD in patients afflicted by chronic ischemic heart disease.
